# Case of Imperforate Anus

**Published:** 1882-07

**Authors:** Herman Mynter


### Clinical Reports.

### Case of Imperforate Anus.

Reported by Herman Mynter, M. D.

Johanne Falk, born November 20th, 1880, was brought to my office June 9th, 1882. At birth, as far as could be learned, no anus was found, but through a recto-vaginal fistula the child was able to have fluid evacuations. As it grew older, more and more trouble was experienced, and when seven months old, the family physician opened the pouch of the rectum by a linear incision. As far as could be learned, no other after-treatment was used than introducing the finger into the wound to prevent contraction. The wound healed in the course of six months, but the cicatricial contraction was so great that no benefit was derived from the operation. During the last six months the child has been feeling quite well, but the difficulty of fecal evacuation has increased very much of late. The evacuations have taken place exclusively through the recto-vaginal fistula and only by aid of frequent injections. By examination, the abdomen was seen large, full and hard. By inspection of the perineum a funnel-shaped fistula, about 1½ inches deep, with whitish, hard, cicatricial walls, was found as the result of the former operation.

The fistula was so narrow that only a common-sized probe could be introduced through the deeper part. Behind and just above the hymen a recto-vaginal fistula was seen, through which a large-sized catheter could be introduced into the rectum, but not large enough to pass the little finger. Under chloroform, Drs. Mann, Folwell, Bartow and Davidson being present, the perineal fistula was incised backwards and forwards so much that the index finger could be introduced into the rectum. The pouch of the rectum was found about 1½ inches from the surface, dilated and spacious. The lateral cicatricial walls of the fistula were dissected down to the pouch, and the lateral and posterior wall of the rectum loosened with scissors for about two inches, while the anterior wall (septum recto-vaginal) was not disturbed as by the division of the perineum forwards, the rectal opening of the fistula was brought much nearer the surface and by a suture could be united with the skin. The pouch of the rectum could now easily be united with the loose skin by numerous sutures, those posteriorly espeically including a great deal of tissue. After the operation two fingers could with ease be introduced into the rectum. The wound was covered with borated cotton. The sutures cut through in five days; union by first intention took place in the anterior half of the wound, but in the posterior part the rectum retracted and left about one inch to be healed by granulation. From the fifth day castor oil was given every day, and every morning, for about fourteen days, large, hard, round fecal masses had to be dug out with the finger. The abdomen gradually became softer, the appetite improved, and at last regular evacuation of the bowels took place. In the course of four weeks the child was sent home into the country, and the mother instructed to introduce a large flexible rubber bougie morning and night and keep it there for ½ hour.

July 15th.—The child was brought back for examination. Wound almost healed; no visible tendency to contraction; bowels move regularly and with ease, and the child has improved much in general health. The recto-vaginal fistula is much smaller and scarcely anything passes through it when the bowels move. The parents were instructed to continue the use of the bougie for an unlimited period. Of the different varieties of imperforate anus, the one in which the rectum opens into the vagina is the least serious, as the child generally suffers very little inconvenience as long as the evacuations are semi-fluid. But as soon as the excrements begin to become solid, serious consequences occur on account of the distention of the colon with following colitis, and an operation is necessary. The linear incision with introduction of bougies afterwards, is very seldom successful, and it is therefore advisable to resort to the more thorough operation of dissecting the gut loose and uniting it with the skin, a method well described in Holmes's surgery.

In the case reported the Rizzoli method was not carried out to its full extent, as by dividing the perineum forwards the anterior part of the rectum could be united with the skin without being dissected loose from the vagina. The fistula was therefore left undisturbed in the hope that it, as "happens tolerably often," might close of its own accord. Although this has not occurred so far, it has diminished considerably in size, and time will show whether farther operative measures will be necessary to close it perfectly.

